# Supplementary material for: Differential Carbon Catabolite Repression and Hemicellulolytic Ability among Pathotypes of Colletotrichum lindemuthianum against Natural Plant Substrates
Source: J Fungi (Basel). 2024 Jun 5;10(6):406. doi: 10.3390/jof10060406 (PMC11204554; doi:10.3390/jof10060406)
Supplement: Supplementary file 1 [file jof-10-00406-s001.zip › Supplementary Figure S1; Tables S1-S7.pdf]

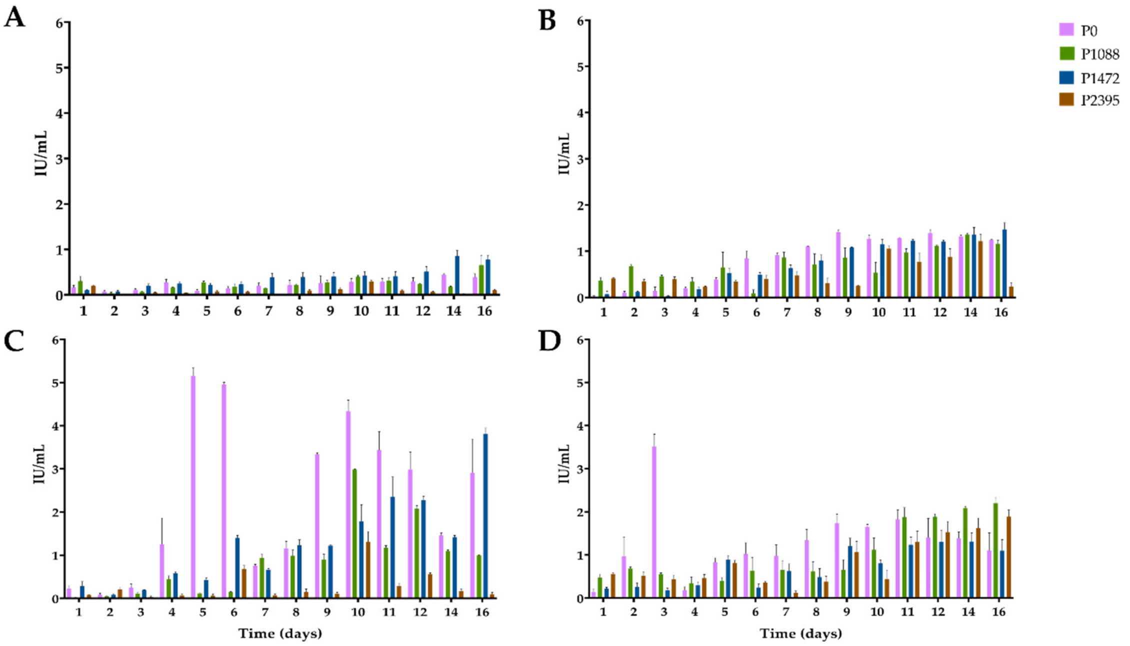

Figure S1. Total protein of pathotypes P0, P1088, P1472 and P2395 of *C. lindemuthianum* in culture with Mathur minimal medium, supplemented with different carbon sources: (A) glucose, (B) hypocotyls, (C) green beans, (D) water hyacinth (*E. crassipes*). Each bar shows the mean of triplicates ± SE.

Table S1. Differential cultivars of common bean (*Phaseolus vulgaris*) currently used for the identification of pathotypes of *Colletotrichum lindemuthianum* proposed by Pastor-Corrales, 1991.

**Num.**	**Differential cultivar**	**Binary value**	**Numeric value (2^{n-1})**	**Resistant genes**	**Gene pool**
1	Michelite	2^0	1	Co-11	MA
2	Michigan Dark Red Kidney	2^1	2	Co-1	A
3	Pery Marrow	2^2	4	$Co\text{-}1^3$	A
4	Cornell 49242	2^3	8	Co-2	MA
5	Widusa	2^4	16	$Co\text{-}1^5$ $Co\text{-}9^3$	A
6	Kaboon	2^5	32	$Co\text{-}1^2$	A
7	Mexico 222	2^6	64	Co-3	MA
8	PI207262	2^7	128	$Co\text{-}4^3$, Co-9, $Co\text{-}3^3$	MA
9	To	2^8	256	Co-4	MA
10	Tu	2^9	512	Co-5	MA
11	AB 136	2^{10}	1024	Co-6, co-8	MA
12	G 2333	2^{11}	2048	$Co\text{-}4^2$, Co-5, Co-7	MA

MA: Mesoamerican gene pool; A: Andean gene pool.

Table S2. Characteristics of the four pathotypes of *Colletotrichum lindemuthianum* used in this study. For more detailed information on pathotypes P0, P1088 and P1472 see González et al. [15]. Pathotype P2395 was kindly provided by Dra. Brenda Z. Guerrero-Aguilar, Dr. José L. Pons-Hernández and Dr. Raul Rodríguez-Guerra from Centro de Investigación Regional del Centro-INIFAP, Celaya, Mexico.

Pathotype	Infected cultivar	Calculation	Virulence index	Collection location	Host bean variety
P0	None	0	0.0	Central Mexico (Michoacan and Jalisco)	Flor de mayo
P1088	Mexico 222 and AB 136	64 + 1024 = 1088	16.6	Northern Mexico (Durango)	Flor de mayo
P1472	Mexico 222, P1207262, To, and AB 136	64 + 128 + 256 + 1024 = 1472	33.3	Central Mexico (Zacatecas, Hidalgo, and Edo. de Mexico)	Flor de mayo
P2395	Michelite, Michigan Dark Red Kidney, Cornell 49242, Widusa, Mexico 222, To, and G2333	1 + 2 + 8 + 16 + 64 + 256 + 2048 = 2395	58.3	Central Mexico (Guanajuato)	Pinto raramuri

Table S3. Statistical analysis of the dry weight of the *C. lindemuthianum* pathotypes and the different substrates analyzed ($p < 0.0001$).

Pathotype	Substrate	Least Sq Mean
0^{A}	Glucose^{C}	54.0767^{G}
	Bean hypocotyls^{ABC}	140.5000^{CD}
	Green beans^{B}	121.0634^{CDE}
	Water hyacinth^{A}	473.9081^{A}
1088^{A}	Glucose^{C}	103.0537^{DEF}
	Bean hypocotyls^{ABC}	155.5000^{C}
	Green beans^{B}	63.3106^{FG}
	Water hyacinth^{A}	163.6075^{C}
1472^{A}	Glucose^{C}	42.7781^{G}
	Bean hypocotyls^{ABC}	151.7391^{CD}
	Green beans^{B}	143.7309^{CD}
	Water hyacinth^{A}	488.1673^{A}
2395^{A}	Glucose^{C}	102.7654^{DEF}
	Bean hypocotyls^{ABC}	145.6352^{CD}
	Green beans^{B}	82.0511^{EFG}
	Water hyacinth^{A}	230.2954^{B}

Values with different letters mean significant differences.

Table S4. Statistical analysis of the XYL activity of the *C. lindemuthianum* pathotypes and the different substrates analyzed ($p < 0.0001$).

Pathotype	Substrate	Least Sq Mean
0^{A}	Glucose^{C}	4.1656^{DE}
	Bean hypocotyls^{B}	8.7186^{CDE}
	Green beans^{B}	10.9673^{BCDE}
	Water hyacinth^{A}	29.3115^{A}
1088^{A}	Glucose^{C}	11.8099^{BCDE}
	Bean hypocotyls^{B}	14.9459^{BC}
	Green beans^{B}	10.5751^{BCDE}
	Water hyacinth^{A}	11.9222^{BCDE}
1472^{A}	Glucose^{C}	2.5721^{E}
	Bean hypocotyls^{B}	13.2991^{BCD}
	Green beans^{B}	7.3939^{CDE}
	Water hyacinth^{A}	36.0558^{A}
2395^{A}	Glucose^{C}	13.1824^{BCDE}
	Bean hypocotyls^{B}	12.7256^{BCDE}
	Green beans^{B}	18.9933^{B}
	Water hyacinth^{A}	14.5594^{BC}

Values with different letters mean significant differences.

Table S5. Statistical analysis of the ABF activity of the *C. lindemuthianum* pathotypes and the different substrates analyzed ($p < 0.0001$).

Pathotype	Substrate	Least Sq Mean
0^{A}	Glucose^{C}	5.6534^{DEFGH}
	Bean hypocotyls^{A}	8.6348^{DEF}
	Green beans^{B}	4.7898^{EFGH}
	Water hyacinth^{B}	5.2102^{EG}
1088^{A}	Glucose^{C}	1.2546^{H}
	Bean hypocotyls^{A}	14.0737^{BC}
	Green beans^{B}	3.4329^{GH}
	Water hyacinth^{B}	10.0109^{CD}
1472^{A}	Glucose^{C}	5.0655^{DEFGH}
	Bean hypocotyls^{A}	8.4597^{DEF}
	Green beans^{B}	5.2753^{DEFGH}
	Water hyacinth^{B}	9.2817^{DF}
2395^{A}	Glucose^{C}	0.8391^{H}
	Bean hypocotyls^{A}	14.1454^{B}
	Green beans^{B}	22.8394^{A}
	Water hyacinth^{B}	8.5718^{DEF}

Values with different letters mean significant differences.

Table S6. Statistical analysis of the XYLO activity of the *C. lindemuthianum* pathotypes and the different substrates analyzed ($p < 0.0001$).

Pathotype	Substrate	Least Sq Mean
0^{A}	$Glucose^{C}$	1.2372^{EFG}
	Bean $hypocotyls^{B}$	8.9649^{BC}
	Green $beans^{B}$	4.7898^{DE}
	Water $hyacinth^{A}$	9.7579^{B}
1088^{A}	$Glucose^{C}$	0.2439^{G}
	Bean $hypocotyls^{B}$	1.3179^{EFG}
	Green $beans^{B}$	2.9898^{DEF}
	Water $hyacinth^{A}$	2.8398^{DEF}
1472^{A}	$Glucose^{C}$	0.7652^{FG}
	Bean $hypocotyls^{B}$	4.7749^{DE}
	Green $beans^{B}$	5.2753^{CD}
	Water $hyacinth^{A}$	13.7487^{A}
2395^{A}	$Glucose^{C}$	0.7848^{FG}
	Bean $hypocotyls^{B}$	1.6162^{DEFG}
	Green $beans^{B}$	3.1726^{DEF}
	Water $hyacinth^{A}$	2.0714^{EFG}

Values with different letters mean significant differences.

Table S7. Statistical analysis of the CBH activity of the *C. lindemuthianum* pathotypes and the different substrates analyzed ($p < 0.0001$).

Pathotype	Substrate	Least Sq Mean
0^{A}	$Glucose^{C}$	0.2229^{F}
	Bean $hypocotyls^{AB}$	6.2316^{BCD}
	Green $beans^{A}$	6.6762^{ABCD}
	Water $hyacinth^{B}$	5.0123^{CD}
1088^{A}	$Glucose^{C}$	0.4599^{F}
	Bean $hypocotyls^{AB}$	6.5367^{ABCD}
	Green $beans^{A}$	9.2792^{A}
	Water $hyacinth^{B}$	5.2061^{CD}
1472^{A}	$Glucose^{C}$	0.4912^{F}
	Bean $hypocotyls^{AB}$	5.1428^{CD}
	Green $beans^{A}$	6.0621^{BCD}
	Water $hyacinth^{B}$	9.0433^{AB}
2395^{A}	$Glucose^{C}$	1.6803^{EF}
	Bean $hypocotyls^{AB}$	7.4932^{ABC}
	Green $beans^{A}$	6.4323^{BCD}
	Water $hyacinth^{B}$	3.7666^{DE}

Values with different letters mean significant differences.
