# Supplementary material for: Differential Carbon Catabolite Repression and Hemicellulolytic Ability among Pathotypes of Colletotrichum lindemuthianum against Natural Plant Substrates
Source: J Fungi (Basel). 2024 Jun 5;10(6):406. doi: 10.3390/jof10060406 (PMC11204554; doi:10.3390/jof10060406)
Supplement: Supplementary file 1 [file jof-10-00406-s001.zip › Supplementary Tables S8 and S9.pdf]

**Supplementary Table S8.** Plant-cell-wall-degrading enzymes secreted by the four pathotypes. The enzymes detected for each pathotype are indicated with an X.

| Protein ID | Enzyme | Molecular weight (kDa) | Family CAZy | P0 | P1088 | P1472 | P2395 |
|---|---|---|---|---|---|---|---|
| A0A135V321;A0A066X3U1;A0A4U6XPW5;H1VI73;A0A1G4AQ83;A0A166S6H7;A0A161VS90;A0A135UAT0;A0A135T1V2;A0A010QG26;A0A5Q4BRG8;A0A4T0VG82;A0A1B7YFG5 | Mannan endo-1,4-beta-mannosidase | 29.135 | GH5 | X | X | | X |
| A0A010RJ28;A0A135TE72;A0A135TKG4;A0A135TU73;A0A1G4B719;A0A4R8QBY1;A0A4R8TA39;N4V5J4;A0A8S9CKF0;A0A4T0VYE1;A0A166PJT8;A0A161YAE7;L2G041;H1V1P3;A0A8H4FHY0;A0A8H3VWS1;A0A4U6XM19;A0A1Q8RSD9;A0A4R8RLN1 | Beta-xylanase | 40.797 | GH10 | X | X | X | X |
| A0A066X1W0 | Glycosyl hydrolase family 16 | 31.952 | GH16 | X | | | |
| A0A066X2B5 | Beta-glucanase | 31.442 | GH16 | X | | | |
| A0A4U6WY38;A0A4U6X097;A0A8H4FFT2;A0A1B7YX19;A0A8H6KVP2;A0A8H6K9G5;A0A8H6IV90;L2GDY3;E3Q936;A0A8S9DJG6;A0A8H3VVM8;A0A5Q4BG50;A0A4V3I574;A0A4T0WC73;A0A4R8RJW7;A0A4R8QGN8;N4VJQ5;T0KUQ1;H1V470;A0A066XIR5 | Alpha-L-arabinofuranosidase C | 4.6282 | GH51 | | X | X | |
| A0A166WB79 | Beta-xylanase | 44.349 | GH10/CBM1 | X | X | X | X |
| A0A8H6NGI3;A0A1G4AVL3;A0A4R8REB9;A0A4R8T6G2;A0A4V3HQA0;A0A8H6IRN5;A0A8H6J316;N4V774;A0A1C6ZZN8;A0A4R8R2A1;A0A166ZDG5;N4VM17;A0A8H6ISX8;A0A0A1I637;A0A8H6MZL9;A0A161VYZ9;A0A8S9DHJ1;A0A4R8PS40;A0A8H6K9R9;A0A4R8TTG4;A0A8H4FPI5;A0A8H3WDA0;T0MBU2;A0A1S1W5J9;A0A135STW0;A0A135S867;A0A010S4E2;A0A135SG86;L2G4N5 | Endo-1,4-beta-xylanase | 23.774 | GH11 | X | X | X | X |
| N4VLB2;A0A4V3HSR7;A0A4R8QG14;A0A1L2S4I5;A0A4R8T7C0 | Endo-beta-1,6-galactanase | 53.339 | GH30 | X | X | X | |
| A0A4R8TG23;A0A4R8QVY5;A0A4R8PZX7;A0A484F6J5;A0A8H6NES4;A0A8H6MYD4;A0A8H6ISR9;T0KGT6;A0A8H4CL91;L2FF07;A0A8S9CJ13;A0A8H3W5L8;A0A7J6IPA5 | Rhamnogalacturonan acetylesterase | 30.95 | CE12 | X | | X | X |
| A0A4R8RFN2;A0A4R8Q8M5;A0A484FCY2;A0A4R8TQT2 | Carboxylic ester hydrolase | 57.795 | CE1 | X | | X | |
| A0A484FDA2;A0A4R8TBC4;A0A4R8QHV8;A0A4T0VPW3;A0A5Q4BJK9;A0A1B7XY52;A0A8H6JVD7;A0A1Q8S6N5;A0A162NTR1;A0A166N5A4;T0L7I6;A0A8H4CBX1;H1UWM6;A0A8H6IQR7;A0A8H6MQW2;A0A8S9CKM5;A0A8H3ZR51;L2FWN2;A0A4V3HWV9 | Beta-galactosidase | 72.507 | GH2 | X | X | X | X |
| A0A484FMV9;A0A4R8QDQ1;A0A4V3I1N1;A0A4R8Q672 | Beta-xylanase | 37.383 | GH10 | X | X | X | X |
| A0A4R8RFP9;A0A4R8TJS2;A0A4V6QEC3;A0A484FPN5;A0A010S9L2;A0A8H6MYU2;A0A135TP44;A0A135V713;A0A1Q8RR97;A0A8H6KUV1;A0A8H6NQH4;T0KL89;A0A166Y6U5;A0A7J6JDL5;A0A8H3ZW79;A0A4U6XR96;A0A4T0W2G0;A0A1S1VBP9;A0A161WMB0;A0A1B7YHG7;A0A8H4CHX7;A0A8S9CQR5;L2FIA5 | Rhamnogalacturonan acetylesterase RhgT | 27.57 | CE12 | X | X | X | X |
| A0A484G3U1;A0A4R8RIN8;A0A4R8Q1T0;A0A4R8TTR1;A0A135UFS3;A0A135TY17;A0A8H3ZVP1;A0A167ANS0;A0A135U033;A0A1G4AQ33;A0A010QDV3 | Beta-galactosidase | 106.92 | GH35 | X | X | X | X |
| A0A4R8TPJ3;A0A4R8PMM5;A0A4R8RQI2;N4VE59 | Pectate lyase C | 32.639 | PL1 | X | X | X | |
| N4V7D8;A0A4R8TLV0;A0A4R8RXP7;A0A4R8PNA1 | Beta-glucosidase 1B | 53.99 | GH1 | | | X | |

| | | | | | | | |
|---|---|---|---|---|---|---|---|
| N4V322;A0A4R8T9L8;A0A4R8PV10;A0A8H6NEE3;A0A8H6KAR8;A0A8H6IR89;A0A4R8PN07;A0A166QMT8;E3QHK2;H1VBK3;A0A4T0VN11;A0A1S1VPQ7;A0A1B7Y056;A0A066XDX7;A0A4U6XI14;A0A166QLQ5 | Cellulase | 46.801 | GH5 | X | | | X |
| Q9Y892;N4W5M1;A0A4R8T5H3;A0A4R8PVB7;A0A4R8QZ27;A0A1S1VG21;A0A8H6J8P7;A0A8H6J7A1;A0A167AIU7 | Pectate lyase 1 | 34.912 | PL1 | X | | | X |
| A0A4R8PXB8;A0A4V3I351;A0A4R8RHT4;N4UZJ3 | Endo-1,3(4)-beta-glucanase | 76.783 | GH16 | X | X | X | X |
| A0A4R8PXH4;A0A4R8T5E5;N4W3K3;A0A4R8RCD5;L2FD33;H1V7Z7;A0A8H3WQ86;A0A8H4FD20;A0A8S9CG29;A0A1B7YC01;A0A8S9D012;A0A8H4FMK3;A0A8H3ZMU2;A0A7J6J365;A0A1G4AYG9;A0A135V9H7;T0LRX0;A0A135SEL4;A0A010SI09;A0A1Q8S4F8;A0A135TS79;A0A166U563;E3Q7R7;A0A066XNW0;A0A161VFA5;L2FW20;A0A1S1VKH8;A0A7J6JPU8 | Beta-glucosidase | 82.186 | GH3 | X | X | X | X |
| A0A4R8QQ58;A0A4R8Q5S5;A0A4R8PXV3;A0A1S1VX18;A0A8H3WMB3;T0KVE5;L2FKB4;A0A8H4FGL4;A0A4U6XIH3;A0A162N4I3;A0A8H6KDN9;A0A8H6JKG7;A0A8H6IVY6;A0A8S9CYJ9;A0A7J6IP67 | Endo-1,4-beta-xylanase B | 28.388 | GH11 | X | | | |
| A0A4R8PZA4;A0A4R8TBK3;N4W2E7;A0A135SC15;A0A135TRC8;A0A135U477;A0A4R8QUR3;A0A010QV31;A0A8H6N8Y6;T0KVB3;L2FTM4;A0A8S9CM83;A0A8H4FKS4;A0A8H3ZHH3;A0A8H6IS29;A0A8H6MX46;A0A5Q4BXB0;A0A1B7YTE8;A0A166WD23;A0A4T0WG78;A0A167BHQ4;A0A1S1VM85;A0A066XB03;A0A1G4AVM1;A0A1Q8RUT7;H1V379;A0A4U6XNC3 | Mannan endo-1,4-beta-mannosidase | 44.961 | GH5 | X | X | X | X |
| A0A4R8T6R7;A0A4R8PZQ0;N4VLI8;A0A135V9H5;A0A135TRE4;A0A135TLV0;A0A010RNV3 | Beta-xylanase | 44.314 | GH10/CBM1 | X | X | X | X |
| N4VIN1;A0A4R8RAV4;A0A4R8Q0D2;A0A4R8T6L5;A0A8H6NZ80;A0A161YE55 | Arabinogalactan endo-beta-1,4-galactanase | 37.707 | GH53 | X | | X | |
| N4VFY8;A0A4R8TC12;A0A4R8RH40;A0A4R8Q692 | Endo-1,4-beta-xylanase | 23.968 | GH11 | X | | X | |
| A0A4R8TK34;A0A4R8Q7H9;A0A8H6NXM0;A0A8H6KHA3;A0A8H6JEQ9;A0A4R8QVN9 | Mannan endo-1,4-beta-mannosidase | 47.034 | GH5/CMB1 | X | X | | |
| N4VFA4;A0A4R8TFZ3;A0A4R8R8H3;A0A4R8QA77;A0A066X9M0 | Glucanase | 49.631 | GH6 | X | X | X | |
| A0A4V3HSH7;A0A4R8QCR4;A0A4R8TJS0;N4V4B6;A0A166QZ17;A0A166SSQ1;L2FF72;A0A8S9DC23;A0A8H4CFS0;A0A2K9RB69;H1V0Z7;T0KDD2;A0A4U6WZL9;A0A1B7Y4W2;A0A5Q4BSE2;A0A4T0VLQ0;A0A1Q8RQ54 | Endopolygalacturonase 1 | 36.662 | GH28 | X | X | X | |
| N4VIP8;A0A4R8TEF2;A0A4R8QE95 | Alpha-galactosidase | 35.611 | GH114 | X | | X | |
| N4V823;A0A4R8TSK7;A0A4R8QF55;A0A4R8RP26;A0A1Q8RP95;A0A135UQ57;A0A8H6NJA6;A0A135S787;A0A8H6MS05;A0A1B7YSJ9;A0A4T0WDS9;H1VQX8;A0A166R2S5;A0A5Q4BKF7;A0A162N2B6;A0A1Q8RU90;A0A7J6IEI2;A0A8S9CRS9;A0A4U6XB84;A0A8H6KRJ0;A0A2Z5DI18;E3QYJ2;A0A8H3ZUS0;A0A484G5W8;A0A4R8RHA6;A0A4R8T639;A0A8S9CZX0;A0A7J6IYC4;A0A8H3WHH6;L2FKU8;T0KH50;L2FY65;A0A8H4FPQ8;A0A8H6JIJ2;A0A8H6NPS3;A0A010S105;A0A135UH76;A0A1G4ATC4;A0A8H4FL70;A0A066XD85;A0A8H6K8V9 | Glucanase | 56.275 | GH7 | X | X | | X |
| N4VJC4;A0A4R8QFE3;A0A4R8RCS2;A0A4R8TU07;A0A8H4CQF0;L2G5M9;T0K8G0;A0A8H6NQ81;A0A7J6J3U8;A0A8H3W888;A0A8S9CS81;A0A8H6KS38;A0A1B7YRS1;A0A4U6WZB0;A0A1S1VBD4;A0A1Q8RT21;A0A5Q4BYI8;H1V7S0;E3QPM6;A0A8H6MU18;A0A4T0W8A1 | Beta-glucosidase | 94.835 | GH3 | X | X | X | X |

| | | | | | | | |
|---|---|---|---|---|---|---|---|
| A0A4R8QGM7;A0A4R8TDK7;A0A4V3HVS7;N4ULE9;A0A4V4NA91;L2FYB4;A0A8S9CDA2;A0A8H4CND0;A0A8H3WTJ5;A0A135UC32;A0A7J6ILT1;A0A8H6NIG2;A0A8H6NEX0;A0A8H6MLK8;A0A5Q4BCF4;A0A1S1VJ28;A0A4U6XSI4;H1VDJ1;A0A1Q8S0P9;A0A1G4BDZ1;A0A166P3D7;A0A161YMM2;A0A135T0F0;A0A135SC94;A0A066XBV4;A0A010RB32;E3QZU3 | Rhamnogalacturonate lyase | 57.652 | PL4 | X | X | X | X |
| N4VBQ3;A0A4R8T7H6;A0A4R8QJV8 | Non-reducing end alpha-L-arabinofuranosidase BoGH43A | 62.164 | GH43 | X | | | |
| N4VF08;A0A4R8TA82;A0A4R8RPX3;A0A4R8QKR1 | Endopolygalacturonase D | 39.54 | GH28 | X | X | X | |
| A0A4R8QLL4;A0A4V3HRF1;A0A4R8TC55;N4VL95;A0A1S1W084;A0A167E5U2;T0KP99;A0A166TDK7 | Pectin lyase F-1 | 50.781 | PL1 | X | | X | |
| A0A4R8RKH5;A0A4R8TGP6;N4VJ71;A0A4R8QM79;A0A8H6U7I2;A0A135RUQ2;A0A166SCX5;A0A5Q4BCI7;A0A4U6XS62;A0A4T0VFU0;A0A010QXQ7;A0A166LBX4;A0A135THW5;A0A135UZ03;A0A8H6NEI4;A0A1S1W3D6;A0A1B7YGJ0;A0A8H6IZT7;A0A1Q8S0N8 | Extracellular exo-alpha-(1->5)-L-arabinofuranosidase | 35.796 | GH43 | X | X | X | X |
| A0A4V6QE96;A0A4R8T957;A0A4R8QP22;N4VHA3;A0A4U6X2K9;A0A4T0VJG7;A0A1B7Y5H6;A0A5Q4BDH2;A0A8H6NJ44;A0A8H6K0S2;A0A8H6IS14;A0A1S1VN04;A0A166R0U4 | Cellobiose dehydrogenase | 89.131 | AA3_1/CBM1 | X | X | X | X |
| N4VND7;A0A4V3I3C3;A0A4R8RR82;A0A4R8QRQ6 | Alpha/beta-glucosidase agdC | 110.58 | GH31 | | | X | |
| A0A4R8RDV5;A0A4V3HTD2;A0A161YKW3 | Pectate lyase B | 34.412 | PL1 | X | X | X | X |
| N4VC67;A0A4V3HSN5;A0A4R8T7T5;A0A4R8RN28;A0A1Q8RSY5;A0A8H6NY40;A0A8H6NLZ8;A0A8H6MV18 | Glycoside hydrolase 131 | 39.357 | GH131 | X | X | X | |
| A0A4R8RPH2;A0A4R8QFB5;A0A4R8TRT8;N4VA40;A0A135T5M9;A0A1Q8S619;A0A1G4ASG4;A0A4U6X672;A0A010QLD6;A0A135SEE3;L2G9P1;A0A135U0H8;T0LNE7;A0A7J6IPZ1;A0A8H3WHB4;A0A8H4CY56;A0A8S9CYP8;A0A5Q4BUF0 | Cellobiose dehydrogenase | 58.69 | AA3_1 | X | | X | X |
| A0A4R8T924;A0A4R8QR39;A0A4R8QE67;N4V855;A0A135UVL0;A0A135RT02;A0A010SKX4;A0A1G4B1Q5;E3QBQ1;A0A1S1VBY5;A0A066XFA8;A0A166QJR5;A0A166MMK8 | Alpha/beta-glucosidase agdC | 104.69 | GH31 | X | X | X | X |
| N4VBS7;A0A4R8TTY4;A0A4V3HWF9;A0A4R8QJC5;A0A8H6IQQ8;L2FQC3 | Xyloglucanase | 87.335 | GH74/CBM1 | X | X | X | X |
| A0A4U6XGA4 | Pectate lyase B | 33.106 | PL1 | X | X | X | X |
| A0A5Q4BHN7 | Beta-xylanase | 45.11 | GH10 | X | | | |
| A0A6M2YG54;A0A484FES1;A0A4R8QD03;A0A4R8TAU8;A0A4R8QN48;A0A1B7XY17;H1V1A9;A0A135U333;A0A4T0VNB6;A0A010RYJ0;A0A135SDQ7;A0A135TXQ8;A0A166N550;A0A5Q4BGB2;A0A8H6MRE0;A0A8H6K8I3;A0A4U6XST8;A0A066X8Q9;A0A1Q8S722;A0A1G4BA36;A0A162NT72;A0A8H6NET6;E3QKA6;A0A8H4CBW9;A0A8H3ZDY6;T0JYM2;L2FVP9;A0A8S9CN22 | Non-reducing end alpha-L-arabinofuranosidase | 72.333 | GH51 | X | X | X | X |
| L2GGL6;A0A8S9CD17;A0A7J6ILC6;T0L0X5;A0A8H3WLE9;A0A8H4CDL0 | Rhamnogalacturonate lyase | 58.652 | PL4 | X | X | X | X |
| A0A7J6JAZ4;T0L7Y9;L2FTU2;A0A8S9CJU9;A0A8H4CSA4;A0A8H3ZM65 | Cellobiose dehydrogenase | 85.825 | AA3_1 | X | X | | |
| A0A8H6K8E0;A0A8H6K3P6;A0A8H6N220;A0A1B2U6V9;T0L420;Q00893;O59939;L2FPZ4;D3YII4;A0A8S9D8V9;A0A8H4C5V8;A0A8H3WCA8;A0A2K9RBA6;Q4PNZ3;I7BL26 | Pectate lyase | 34.221 | PL1 | X | X | X | X |
| A0A8H6KHC7;A0A8H6MPX5;A0A8H6KWL4 | Beta-xylanase | 51.063 | GH10/CBM1 | X | | X | |
| A0A8S9DJL4;L2GIL4;A0A8H3ZQ94 | Beta-xylanase | 37.288 | GH10 | X | X | X | |
| N4UVD6;A0A4R8TSD5;A0A1Q8RRQ1 | Pectate lyase B | 34.382 | PL1 | X | | X | |

| | | | | | | | |
|---|---|---|---|---|---|---|---|
| N4V8X1 | Mannan endo-1,4-beta-mannosidase | 51.798 | GH26 | X | X | X | X |
| N4VCV9;A0A4R8QB16;A0A4R8RFL6;A0A167DE51;A0A1S1VW33;A0A161YPW9;A0A8H6NBR0;A0A8H6ISN8;A0A135UXV9;A0A8H6N8W4;A0A8H6MMQ4;A0A8H6J7R1;A0A135T8F8;A0A1S1VIM1;A0A166U483;A0A4T0W753;A0A010QW28;A0A161VF97;A0A8H6KI97;A0A5Q4BIK0;H1VP94;A0A8H6N085;A0A8H6K8F3;A0A135V9X4;A0A135RS48;E3QPU6;A0A8S9CEF9;A0A8H3W8I2;A0A135S938;A0A8H6K4Y5;A0A066XN55;A0A7J6IHZ9;A0A8H4CDF8 | Carboxylic ester hydrolase | 52.333 | CE1 | X | X | X | X |
| N4VDM2;A0A4R8QB94;A0A1Q8RBU4;A0A161YMG1;E3Q3Q4;A0A066XDL8;A0A4R8T363;A0A4R8RFP8 | Alpha-L-rhamnosidase rgxB | 49.857 | GH28 | X | | | X |
| Q00446;A0A8H3ZJ79 | Endopolygalacturonase 1 | 36.712 | GH28 | X | X | X | |
| A0A484FL16;A0A135SIW6;A0A135UN15;A0A135TLC4;A0A4R8RNW8;A0A010RHD3;A0A4R8S9Y7;A0A4R8QEL8;E3Q317;T0K4W2;A0A8H4CM13;A0A166VB31;A0A1S1VNL1;A0A8H3WIX8;L2G9R4;A0A162N686;A0A8S9D1C0;A0A7J6JPR9;A0A1Q8RVD9;A0A5Q4C173;A0A1G4B254;A0A4U6X6S0;A0A4T0VCG6;A0A1B7XX47;A0A8H3ZUD5;A0A7J6JMB6;A0A8S9CJ70;A0A8H4C884;H1UYG4;A0A8H6MSA5;A0A8H6NWF6;H1V1U8;H1VHR6;A0A4V6DHQ5;A0A4T0WB56;A0A066XX88;A0A8H6NNU0;L2GBW1;T0LLN5 | Glycoside hydrolase 131 | 28.703 | GH131 | X | | X | |
| A0A1Q8S953;A0A8H6JK50;A0A135UU87;A0A8H6ITA7;A0A8H6NJX7;A0A4V3HS25;A0A4V3I1R0;N4V576;A0A4R8QKK9;A0A010S7Z8;T0MBC6;A0A135TJM9;A0A135TH29;A0A1S1W0B5;A0A8H3WMG5;A0A8H4CDT0;A0A8S9C7N1;L2GAY0;A0A161WDP5 | Glycoside hydrolase 131 | 32.067 | GH131 | | | X | |
| A0A1B7XWZ2;A0A4T0VDY3;A0A4U6XHT9;A0A5Q4BFE3;A0A1Q8S5K2;A0A161WIK2;A0A166PA54;T0KZM4;A0A135SIJ4;A0A1G4BQX2;A0A135RPD8;A0A010QZF0;A0A8H4CQA6;A0A8H6J7R8;A0A8H6KQC9;A0A8H6NJN2;A0A8S9DI55;A0A7J6J8V6;A0A8H3ZTK9;A0A135V2V6 | Glucanase | 50.605 | GH6/CMB1 | X | | | |

**Supplementary Table S9**. Secretion and differential regulation of PCWDEs from *C. lindemuthianum* pathotypes in glucose and green bean cultures. Upregulated enzymes for each pathotype are indicated with an X.

| **Protein ID** | **Enzyme** | **Molecular weight (kDa)** | **Family CAZy** | **P0** | **P1088** | **P1472** | **P2395** | **P0** | **P1088** | **P1472** | **P2395** |
|---|---|---|---|---|---|---|---|---|---|---|---|
| | | | | **Glucose** | | | | **Green beans** | | | |
| A0A135V321;A0A066X3U1;A0A4U6XPW5;H1VI73;A0A1G4AQ83;A0A166S6H7;A0A161VS90;A0A135UAT0;A0A135T1V2;A0A010QG26;A0A5Q4BRG8;A0A4T0VG82;A0A1B7YFG5 | Mannan endo-1,4-beta-mannosidase | 29.135 | GH5 | | X | | | X | X | | X |
| A0A484FL16;A0A135SIW6;A0A135UN15;A0A135TLC4;A0A4R8RNW8;A0A010RHD3;A0A4R8S9Y7;A0A4R8QEL8;E3Q317;T0K4W2;A0A8H4CM13;A0A166VB31;A0A1S1VNL1;A0A8H3WIX8;L2G9R4;A0A162N686;A0A8S9D1C0;A0A7J6JPR9;A0A1Q8RVD9;A0A5Q4C173;A0A1G4B254;A0A4U6X6S0;A0A4T0VCG6;A0A1B7XX47;A0A8H3ZUD5;A0A7J6JMB6;A0A8S9CJ70;A0A8H4C884;H1UYG4;A0A8H6MSA5;A0A8H6NWF6;H1V1U8;H1VHR6;A0A4V6DHQ5;A0A4T0WB56;A0A066XX88;A0A8H6NNU0;L2GBW1;T0LLN5 | Glycoside hydrolase 131 | 28.703 | GH131 | X | | X | | | | | |
| A0A010RJ28;A0A135TE72;A0A135TKG4;A0A135TU73;A0A1G4B719;A0A4R8QBY1;A0A4R8TA39;N4V5J4;A0A8S9CKF0;A0A4T0VYE1;A0A166PJT8;A0A161YAE7;L2G041;H1V1P3;A0A8H4FHY0;A0A8H3VWS1;A0A4U6XM19;A0A1Q8RSD9;A0A4R8RLN1 | Beta-xylanase | 40.797 | GH10 | | | | | X | X | X | X |
| A0A1Q8S953;A0A8H6JK50;A0A135UU87;A0A8H6ITA7;A0A8H6NJX7;A0A4V3HS25;A0A4V3I1R0;N4V576;A0A4R8QKK9;A0A010S7Z8;T0MBC6;A0A135TJM9;A0A135TH29;A0A1S1W0B5;A0A8H3WMG5;A0A8H4CDT0;A0A8S9C7N1;L2GAY0;A0A161WDP5 | Glycoside hydrolase 131 | 32.067 | GH131 | | | X | | | | | |
| A0A066X1W0 | Glycosyl hydrolase family 16 | 31.952 | GH16 | | | x | | x | | x | x |
| A0A066X2B5 | Beta-glucanase | 31.442 | GH16 | | | | | x | | x | |
| A0A4U6WY38;A0A4U6X097;A0A8H4FFT2;A0A1B7YX19;A0A8H6KVP2;A0A8H6K9G5;A0A8H6IV90;L2GDY3;E3Q936;A0A8S9DJG6;A0A8H3VVM8;A0A5Q4BG50;A0A4V3I574;A0A4T0WC73;A0A4R8RJW7;A0A4R8QGN8;N4VJQ5;T0KUQ1;H1V470;A0A066XIR5 | Alpha-L-arabinofuranosidase C | 4.6282 | GH51 | | X | X | | | | | |
| A0A166WB79 | Beta-xylanase | 44.349 | GH10/CBM1 | | X | | X | X | X | X | X |

| | | | | | | | | | | | |
|---|---|---|---|---|---|---|---|---|---|---|---|
| A0A1B7XWZ2;A0A4T0VDY3;A0A4U6XHT9;A0A5Q4BFE3;A0A1Q8S5K2;A0A161WIK2;A0A166PA54;T0KZM4;A0A135SIJ4;A0A1G4BQX2;A0A135RPD8;A0A010QZF0;A0A8H4CQA6;A0A8H6J7R8;A0A8H6KQC9;A0A8H6NJN2;A0A8S9DI55;A0A7J6J8V6;A0A8H3ZTK9;A0A135V2V6 | Glucanase | 50.605 | GH6/CBM1 | | | | | X | | | |
| A0A8H6NGI3;A0A1G4AVL3;A0A4R8REB9;A0A4R8T6G2;A0A4V3HQA0;A0A8H6IRN5;A0A8H6J316;N4V774;A0A1C6ZZN8;A0A4R8R2A1;A0A166ZDG5;N4VM17;A0A8H6ISX8;A0A0A1I637;A0A8H6MZL9;A0A161VYZ9;A0A8S9DHJ1;A0A4R8PS40;A0A8H6K9R9;A0A4R8TTG4;A0A8H4FPI5;A0A8H3WDA0;T0MBU2;A0A1S1W5J9;A0A135STW0;A0A135S867;A0A010S4E2;A0A135SG86;L2G4N5 | Endo-1,4-beta-xylanase | 23.774 | GH11 | X | X | X | | X | X | X | X |
| N4VLB2;A0A4V3HSR7;A0A4R8QG14;A0A1L2S4I5;A0A4R8T7C0 | Endo-beta-1,6-galactanase | 53.339 | GH30 | X | X | X | | X | | X | X |
| A0A4R8TG23;A0A4R8QVY5;A0A4R8PZX7;A0A484F6J5;A0A8H6NES4;A0A8H6MYD4;A0A8H6ISR9;T0KGT6;A0A8H4CL91;L2FF07;A0A8S9CJ13;A0A8H3W5L8;A0A7J6IPA5 | Rhamnogalacturonan acetylesterase | 30.95 | CE12 | X | | X | X | | | | |
| A0A4R8RFN2;A0A4R8Q8M5;A0A484FCY2;A0A4R8TQT2 | Carboxylic ester hydrolase | 57.795 | CE1 | X | | X | | | | | |
| A0A484FDA2;A0A4R8TBC4;A0A4R8QHV8;A0A4T0VPW3;A0A5Q4BJK9;A0A1B7XY52;A0A8H6JVD7;A0A1Q8S6N5;A0A162NTR1;A0A166N5A4;T0L7I6;A0A8H4CBX1;H1UWM6;A0A8H6IQR7;A0A8H6MQW2;A0A8S9CKM5;A0A8H3ZR51;L2FWN2;A0A4V3HWV9 | Beta-galactosidase | 72.507 | GH2 | X | X | X | X | X | X | X | X |
| A0A484FMV9;A0A4R8QDQ1;A0A4V3I1N1;A0A4R8Q672 | Beta-xylanase | 37.383 | GH10 | X | X | X | X | | | | |
| A0A4R8RFP9;A0A4R8TJS2;A0A4V6QEC3;A0A484FPN5;A0A010S9L2;A0A8H6MYU2;A0A135TP44;A0A135V713;A0A1Q8RR97;A0A8H6KUV1;A0A8H6NQH4;T0KL89;A0A166Y6U5;A0A7J6JDL5;A0A8H3ZW79;A0A4U6XR96;A0A4T0W2G0;A0A1S1VBP9;A0A161WMB0;A0A1B7YHG7;A0A8H4CHX7;A0A8S9CQR5;L2FIA5 | Rhamnogalacturonan acetylesterase RhgT | 27.57 | CE12 | X | X | X | X | X | X | X | X |
| A0A484G3U1;A0A4R8RIN8;A0A4R8Q1T0;A0A4R8TTR1;A0A135UFS3;A0A135TY17;A0A8H3ZVP1;A0A167ANS0;A0A135U033;A0A1G4AQ33;A0A010QDV3 | Beta-galactosidase | 106.92 | GH35 | X | X | X | X | | | | |
| A0A4R8TPJ3;A0A4R8PMM5;A0A4R8RQI2;N4VE59 | Putative pectate lyase C | 32.639 | PL1 | X | | X | | | X | | |
| N4V7D8;A0A4R8TLV0;A0A4R8RXP7;A0A4R8PNA1 | Beta-glucosidase 1B | 53.99 | GH1 | | | X | | | | | |

| | | | | | | | | | | | |
|---|---|---|---|---|---|---|---|---|---|---|---|
| N4V322;A0A4R8T9L8;A0A4R8PV10;A0A8H6NEE3;A0A8H6KAR8;A0A8H6IR89;A0A4R8PN07;A0A166QMT8;E3QHK2;H1VBK3;A0A4T0VN11;A0A1S1VPQ7;A0A1B7Y056;A0A066XDX7;A0A4U6XI14;A0A166QLQ5 | Cellulase (glycosyl hydrolase family 5) | 46.801 | GH5 | | | | | X | | | X |
| Q9Y892;N4W5M1;A0A4R8T5H3;A0A4R8PVB7;A0A4R8QZ27;A0A1S1VG21;A0A8H6J8P7;A0A8H6J7A1;A0A167AIU7 | Pectate lyase 1 | 34.912 | PL1 | | | | | X | | | X |
| A0A4R8PXB8;A0A4V3I351;A0A4R8RHT4;N4UZJ3 | Endo-1,3(4)-beta-glucanase | 76.783 | GH16 | X | X | X | X | | | | |
| A0A4R8PXH4;A0A4R8T5E5;N4W3K3;A0A4R8RCD5;L2FD33;H1V7Z7;A0A8H3WQ86;A0A8H4FD20;A0A8S9CG29;A0A1B7YC01;A0A8S9D012;A0A8H4FMK3;A0A8H3ZMU2;A0A7J6J365;A0A1G4AYG9;A0A135V9H7;T0LRX0;A0A135SEL4;A0A010SI09;A0A1Q8S4F8;A0A135TS79;A0A166U563;E3Q7R7;A0A066XNW0;A0A161VFA5;L2FW20;A0A1S1VKH8;A0A7J6JPU8 | Beta-glucosidase | 82.186 | GH3 | X | X | X | X | | X | X | |
| A0A4R8QQ58;A0A4R8Q5S5;A0A4R8PXV3;A0A1S1VX18;A0A8H3WMB3;T0KVE5;L2FKB4;A0A8H4FGL4;A0A4U6XIH3;A0A162N4I3;A0A8H6KDN9;A0A8H6JKG7;A0A8H6IVY6;A0A8S9CYJ9;A0A7J6IP67 | Endo-1,4-beta-xylanase B | 28.388 | GH11 | X | | | | X | | | |
| A0A4R8PZA4;A0A4R8TBK3;N4W2E7;A0A135SC15;A0A135TRC8;A0A135U477;A0A4R8QUR3;A0A010QV31;A0A8H6N8Y6;T0KVB3;L2FTM4;A0A8S9CM83;A0A8H4FKS4;A0A8H3ZHH3;A0A8H6IS29;A0A8H6MX46;A0A5Q4BXB0;A0A1B7YTE8;A0A166WD23;A0A4T0WG78;A0A167BHQ4;A0A1S1VM85;A0A066XB03;A0A1G4AVM1;A0A1Q8RUT7;H1V379;A0A4U6XNC3 | Mannan endo-1,4-beta-mannosidase | 44.961 | GH5 | X | X | X | | X | X | X | X |
| A0A4R8T6R7;A0A4R8PZQ0;N4VLI8;A0A135V9H5;A0A135TRE4;A0A135TLV0;A0A010RNV3 | Beta-xylanase | 44.314 | GH10/CBM1 | X | X | X | X | X | X | X | X |
| N4VIN1;A0A4R8RAV4;A0A4R8Q0D2;A0A4R8T6L5;A0A8H6NZ80;A0A161YE55 | Arabinogalactan endo-beta-1,4-galactanase | 37.707 | GH53 | X | | X | | | | | |
| N4VFY8;A0A4R8TC12;A0A4R8RH40;A0A4R8Q692 | Endo-1,4-beta-xylanase | 23.968 | GH11 | X | | X | | | | | |
| A0A4R8TK34;A0A4R8Q7H9;A0A8H6NXM0;A0A8H6KHA3;A0A8H6JEQ9;A0A4R8QVN9 | Mannan endo-1,4-beta-mannosidase | 47.034 | GH5/CMB1 | | | | | X | X | | |
| N4VFA4;A0A4R8TFZ3;A0A4R8R8H3;A0A4R8QA77;A0A066X9M0 | Glucanase | 49.631 | GH6 | | | | | X | X | X | |
| A0A4V3HSH7;A0A4R8QCR4;A0A4R8TJS0;N4V4B6;A0A166QZ17;A0A166SSQ1;L2FF72;A0A8S9DC23;A0A8H4CFS0;A0A2K9RB69;H1V0Z7;T0KDD2;A0A4U6WZL9;A0A1B7Y4W2;A0A5Q4BSE2;A0A4T0VLQ0;A0A1Q8RQ54 | Endopolygalacturonase 1 | 36.662 | GH28 | X | X | X | | | | | |
| N4VIP8;A0A4R8TEF2;A0A4R8QE95 | Alpha-galactosidase | 35.611 | GH114 | X | | X | | | | | |

| | | | | | | | | | | | |
|---|---|---|---|---|---|---|---|---|---|---|---|
| N4V823;A0A4R8TSK7;A0A4R8QF55;A0A4R8RP26;A0A1Q8RP95;A0A135UQ57;A0A8H6NJA6;A0A135S787;A0A8H6MS05;A0A1B7YSJ9;A0A4T0WDS9;H1VQX8;A0A166R2S5;A0A5Q4BKF7;A0A162N2B6;A0A1Q8RU90;A0A7J6IEI2;A0A8S9CRS9;A0A4U6XB84;A0A8H6KRJ0;A0A2Z5DI18;E3QYJ2;A0A8H3ZUS0;A0A484G5W8;A0A4R8RHA6;A0A4R8T639;A0A8S9CZX0;A0A7J6IYC4;A0A8H3WHH6;L2FKU8;T0KH50;L2FY65;A0A8H4FPQ8;A0A8H6JIJ2;A0A8H6NPS3;A0A010S105;A0A135UH76;A0A1G4ATC4;A0A8H4FL70;A0A066XD85;A0A8H6K8V9 | Glucanase | 56.275 | GH7 | X | X | | X | X | X | | |
| N4VJC4;A0A4R8QFE3;A0A4R8RCS2;A0A4R8TU07;A0A8H4CQF0;L2G5M9;T0K8G0;A0A8H6NQ81;A0A7J6J3U8;A0A8H3W888;A0A8S9CS81;A0A8H6KS38;A0A1B7YRS1;A0A4U6WZB0;A0A1S1VBD4;A0A1Q8RT21;A0A5Q4BYI8;H1V7S0;E3QPM6;A0A8H6MU18;A0A4T0W8A1 | Beta-glucosidase | 94.835 | GH3 | X | X | X | X | X | X | X | X |
| A0A4R8QGM7;A0A4R8TDK7;A0A4V3HVS7;N4ULE9;A0A4V4NA91;L2FYB4;A0A8S9CDA2;A0A8H4CND0;A0A8H3WTJ5;A0A135UC32;A0A7J6ILT1;A0A8H6NIG2;A0A8H6NEX0;A0A8H6MLK8;A0A5Q4BCF4;A0A1S1VJ28;A0A4U6XSI4;H1VDJ1;A0A1Q8S0P9;A0A1G4BDZ1;A0A166P3D7;A0A161YMM2;A0A135T0F0;A0A135SC94;A0A066XBV4;A0A010RB32;E3QZU3 | Rhamnogalacturonate lyase | 57.652 | PL4 | X | X | X | | | X | X | X |
| N4VBQ3;A0A4R8T7H6;A0A4R8QJV8 | Non-reducing end alpha-L-arabinofuranosidase BoGH43A | 62.164 | GH43 | X | | | | X | | | |
| N4VF08;A0A4R8TA82;A0A4R8RPX3;A0A4R8QKR1 | Endopolygalacturonase D | 39.54 | GH28 | X | X | X | | | | | |
| A0A4R8QLL4;A0A4V3HRF1;A0A4R8TC55;N4VL95;A0A1S1W084;A0A167E5U2;T0KP99;A0A166TDK7 | Pectin lyase F-1 | 50.781 | PL1 | X | | X | | | | | |
| A0A4R8RKH5;A0A4R8TGP6;N4VJ71;A0A4R8QM79;A0A8H6U7I2;A0A135RUQ2;A0A166SCX5;A0A5Q4BCI7;A0A4U6XS62;A0A4T0VFU0;A0A010QXQ7;A0A166LBX4;A0A135THW5;A0A135UZ03;A0A8H6NEI4;A0A1S1W3D6;A0A1B7YGJ0;A0A8H6IZT7;A0A1Q8S0N8 | Extracellular exo-alpha-(1->5)-L-arabinofuranosidase | 35.796 | GH43 | X | X | X | X | X | | X | X |
| A0A4V6QE96;A0A4R8T957;A0A4R8QP22;N4VHA3;A0A4U6X2K9;A0A4T0VJG7;A0A1B7Y5H6;A0A5Q4BDH2;A0A8H6NJ44;A0A8H6K0S2;A0A8H6IS14;A0A1S1VN04;A0A166R0U4 | Cellobiose dehydrogenase | 89.131 | AA3_1/CBM1 | X | X | | X | X | X | X | X |
| N4VND7;A0A4V3I3C3;A0A4R8RR82;A0A4R8QRQ6 | Alpha/beta-glucosidase agdC | 110.58 | GH31 | | X | X | | | | | |
| A0A4R8RDV5;A0A4V3HTD2;A0A161YKW3 | Pectate lyase B | 34.412 | PL1 | X | X | X | X | X | X | X | X |

| | | | | | | | | | | | |
|---|---|---|---|---|---|---|---|---|---|---|---|
| N4VC67;A0A4V3HSN5;A0A4R8T7T5;A0A4R8RN28;A0A1Q8RSY5;A0A8H6NY40;A0A8H6NLZ8;A0A8H6MV18 | Glycoside hydrolase 131 | 39.357 | GH131 | X | X | X | | | | | |
| A0A4R8RPH2;A0A4R8QFB5;A0A4R8TRT8;N4VA40;A0A135T5M9;A0A1Q8S619;A0A1G4ASG4;A0A4U6X672;A0A010QLD6;A0A135SEE3;L2G9P1;A0A135U0H8;T0LNE7;A0A7J6IPZ1;A0A8H3WHB4;A0A8H4CY56;A0A8S9CYP8;A0A5Q4BUF0 | Cellobiose dehydrogenase | 58.69 | AA3_1 | X | | X | X | | | | |
| A0A4R8T924;A0A4R8QR39;A0A4R8QE67;N4V855;A0A135UVL0;A0A135RT02;A0A010SKX4;A0A1G4B1Q5;E3QBQ1;A0A1S1VBY5;A0A066XFA8;A0A166QJR5;A0A166MMK8 | Alpha/beta-glucosidase agdC | 104.69 | GH31 | X | X | X | X | X | X | X | X |
| N4VBS7;A0A4R8TTY4;A0A4V3HWF9;A0A4R8QJC5;A0A8H6IQQ8;L2FQC3 | Xyloglucanase | 87.335 | GH74/CBM1 | | | | | X | X | X | X |
| A0A4U6XGA4 | Pectate lyase B | 33.106 | PL1 | X | X | X | | | X | X | X |
| A0A5Q4BHN7 | Beta-xylanase | 45.11 | GH10 | | | | | X | | | |
| A0A6M2YG54;A0A484FES1;A0A4R8QD03;A0A4R8TAU8;A0A4R8QN48;A0A1B7XY17;H1V1A9;A0A135U333;A0A4T0VNB6;A0A010RYJ0;A0A135SDQ7;A0A135TXQ8;A0A166N550;A0A5Q4BGB2;A0A8H6MRE0;A0A8H6K8I3;A0A4U6XST8;A0A066X8Q9;A0A1Q8S722;A0A1G4BA36;A0A162NT72;A0A8H6NET6;E3QKA6;A0A8H4CBW9;A0A8H3ZDY6;T0JYM2;L2FVP9;A0A8S9CN22 | Non-reducing end alpha-L-arabinofuranosidase | 72.333 | GH51 | | X | | | X | X | X | X |
| L2GGL6;A0A8S9CD17;A0A7J6ILC6;T0L0X5;A0A8H3WLE9;A0A8H4CDL0 | Rhamnogalacturonate lyase | 58.652 | PL4 | X | X | X | X | | | | |
| A0A7J6JAZ4;T0L7Y9;L2FTU2;A0A8S9CJU9;A0A8H4CSA4;A0A8H3ZM65 | Cellobiose dehydrogenase | 85.825 | AA3 | | | | | X | X | | |
| A0A8H6K8E0;A0A8H6K3P6;A0A8H6N220;A0A1B2U6V9;T0L420;Q00893;O59939;L2FPZ4;D3YII4;A0A8S9D8V9;A0A8H4C5V8;A0A8H3WCA8;A0A2K9RBA6;Q4PNZ3;I7BL26 | Pectate lyase | 34.221 | PL1 | X | X | X | X | | | | |
| A0A8H6KHC7;A0A8H6MPX5;A0A8H6KWL4 | Beta-xylanase | 51.063 | GH10/CBM1 | X | | X | | | | | |
| A0A8S9DJL4;L2GIL4;A0A8H3ZQ94 | Beta-xylanase | 37.288 | GH10 | X | X | X | | | | | |
| N4UVD6;A0A4R8TSD5;A0A1Q8RRQ1 | Pectate lyase B | 34.382 | PL1 | X | | X | | | | | |
| N4V8X1 | Mannan endo-1,4-beta-mannosidase | 51.798 | GH26 | X | X | X | X | | | | |
| N4VCV9;A0A4R8QB16;A0A4R8RFL6;A0A167DE51;A0A1S1VW33;A0A161YPW9;A0A8H6NBR0;A0A8H6ISN8;A0A135UXV9;A0A8H6N8W4;A0A8H6MMQ4;A0A8H6J7R1;A0A135T8F8;A0A1S1VIM1;A0A166U483;A0A4T0W753;A0A010QW28;A0A161VF97;A0A8H6KI97;A0A5Q4BIK0;H1VP94;A0A8H6N085;A0A8H6K8F3;A0A135V9X4;A0A135RS48;E3QPU6;A0A8S9CEF9;A0A8H3W8I2;A0A135S938;A0A8H6K4Y5;A0A066XN55;A0A7J6IHZ9;A0A8H4CDF8 | Carboxylic ester hydrolase | 52.333 | CE1 | X | X | X | X | X | X | X | X |

| | | | | | | | | | | | |
|---|---|---|---|---|---|---|---|---|---|---|---|
| N4VDM2;A0A4R8QB94;A0A1Q8RBU4;A0A161YMG1;E3Q3Q4;A0A066XDL8;A0A4R8T363;A0A4R8RFP8 | Alpha-L-rhamnosidase rgxB | 49.857 | GH28 | X | | | X | | | | |
| Q00446;A0A8H3ZJ79 | Endopolygalacturonase 1 | 36.712 | GH28 | X | X | X | | | | | |
